# Estrogen receptor alpha (ERα) mRNA copy numbers in immunohistochemically ERα-positive-, and negative breast cancer tissues

**DOI:** 10.1186/1471-2407-7-56

**Published:** 2007-03-28

**Authors:** Indira Poola, Qingqi Yue

**Affiliations:** 1 Department of Biochemistry and Molecular Biology, Howard University School of Medicine, Washington, D. C. 20059, USA; 2 Statistical Genetics and Bioinformatics Unit, Howard University School of Medicine, Washington, D. C. 20059, USA

## Abstract

**Background:**

The presence of ERα is the basis for treating breast cancer patients with targeted molecular therapies that block estrogen stimulation of breast cancer cell division. To select patients for the above therapies, currently, the ERα presence in breast cancer tissues is determined in clinical laboratories by microscopically scoring the slides subjected to immunohistochemistry (IHC). This method is not quantitative, highly subjective and requires large amount of tumor tissue, therefore, cannot be applied to sterotactic and ultrasound guided biopsy samples. To circumvent these problems, we previously developed quantitative real-time PCR based molecular assay that can be applied to determine mRNA copies of ERα in picogram amounts of total RNA from tumor samples. However, it is not known how the mRNA copy numbers correlate to IHC positive and negative status.

**Methods:**

In the current study we determined the copy numbers of ERα mRNA by Q RTPCR in breast cancer tissues that were graded as ERα-positive and negative by 1) IHC and 2) functional estrogen binding assay and statistically analyzed the data.

**Results:**

We demonstrate here that ERα mRNA copy numbers are not significantly different in tissues that are graded as positive by IHC and ligand binding assays. We establish here a cut of value of 5 × 10^6 ^copies per 10^10 ^mRNA copies of GAPDH with an Odds Radio of 39.4, Sensitivity of 0.81 and Specificity of 0.90 in breast cancer tissues that are negative for ERα protein by IHC and estrogen binding assays. ROC analysis of the data gave an area of 0.8967 under the curve.

**Conclusion:**

We expect that the cut off values determined here will be highly significant for applying molecular assay in the place of IHC in clinical laboratories for evaluating the presence of ERα for prognostic and therapeutic purposes.

## Background

Breast cancer is the most diagnosed and the second leading cause of cancer deaths for women in the United States striking about 300,000 and killing about 40,000 women a year [1]. A substantial body of epidemiological, experimental and clinical evidence indicated that unopposed stimulation of breast epithelial cells by the natural hormone, estrogen, plays a major role in the progression of breast cancers [2]. Because endogenous estrogens directly promote the growth of breast cancer cells, estrogen deprivation either by inhibiting its biosynthesis (aromatase inhibitors) or blocking estrogen-mediated gene transcription (tamoxifen) through its high affinity receptor, the estrogen receptor alpha (ERα), are the primary lines of therapy for breast cancer patients. In most cases, the efficacy of the above treatments has correlated with the presence of ERα in the tumor tissues. Currently, only those patients who express ERα in their tumors are chosen for aromatase inhibitor or tamoxifen therapies. In addition to being a therapeutic target, ERα was also shown to be the most important factor to predict breast cancer prognosis. The patients who express ERα in their tumors have an overall longer cancer-free survival and lower recurrence rates than patients who do not express this receptor [3].

To predict prognosis and identify patients for the above two anti-estrogen therapies, every breast cancer tissue is currently screened for the presence of ERα before a treatment regimen is selected for any breast cancer patient. The presence of ERα in breast tumors was originally determined in clinical labs by estrogen binding assay for about 20 years. However, when the tumors were detected at comparatively smaller sizes and highly specific monoclonal antibodies were developed that could detect ERα both in the fresh frozen as well as formalin fixed paraffin-embedded tissues, the clinical labs switched to immunohistochemistry (IHC) from estrogen binding assay for determining the presence of ERα. Currently ERα is determined in the clinical laboratories from rough estimates yielded by microscopically scoring the slides subjected to IHC technique using antibodies against the N-terminal A/B region of ERα. Although this procedure is used for over ten years, it has several limitations including not quantitative, highly subjective, variations due to antibody preparations, variations from one clinical lab to other and comparatively large sample size requirement.

In recent times, due to increased awareness and substantially improved screening methods, breast cancers are detected at very early stages and excised, in a large majority of cases, by stereotactic and ultrasound guided techniques. In these cases the limited amount of tumor tissue that remains after histological testing restricts determining ERα status for prognostic and therapeutic purposes by IHC. In many cases, ERα status is not determined due to insufficient amount of tumor tissue. For these reasons there is an urgent need to switch to a procedure that can detect ERα in a very small amount of tumor tissue obtained by the above methods. There is a general consensus that ERα mRNA quantification is a more suited technique for detecting its presence in tumor tissues. Several PCR based approaches have been described for detecting the presence of ERα in breast cancer tissues [4,5]. We recently developed a highly sensitive real-time PCR based quantitative molecular assay that can detect and quantify as low as 50–100 copies of ERα mRNA from as small as 40 picograms of total RNA from breast cancer tissues. Because quantitative real-time PCR is a high through-put method, it could be automated to apply in clinical laboratories. However, it is not known how the ERα mRNA copy numbers correlate to ERα positivity and negativity by IHC assay. Establishing a cut off value in IHC negative tissues is required for the application of molecular assay in the place of IHC assay. To determine the cut off value, we have profiled ERα mRNA copy numbers in breast cancer tissues which have been graded as ERα positive and negative by IHC and estrogen binding assays. We demonstrate here that ERα mRNA copy numbers are not significantly different in tissues that were graded as positive by IHC and ligand binding assays. However, ERα positive tissues, either by IHC or estrogen binding assays, express significantly higher mRNA copy numbers than the negative tissues. We have determined the cut off values of ERα mRNA copy numbers by molecular assay that correlate to ERα negativity by both IHC and ligand binding assays using CART program (Classification And Regression Tree). We expect that the cut off values determined here will be highly significant for applying molecular assay in the place of IHC in clinical laboratories for determining the ERα status for prognostic and therapeutic purposes.

## Methods

All the primers used in the current study were synthesized by Gibco-BRL Life Technologies. TaqMan Universal PCR Master Mix (Cat # 4304437) was from Applied Biosystems. 5'FAM and 3'TAMARA labeled oligonucleotide probes were synthesized by Applied Biosystems and available from previous studies. PCR quality water and Tris-EDTA buffer were from BioWhittaker.

### Breast tumor samples

Breast cancer tissues with known ERα status by IHC and ligand binding assay were available from previous studies [6-9]. Briefly, the tumor samples were collected from either biopsy or mastectomies immediately after surgery and stored at -80°C until use. Fresh tumor tissue samples for ERα quantification were routinely harvested immediately adjacent to the histologic/diagnostic sections and considered to be representative of the tissue used for diagnosis. All the samples were examined by a pathologist and tissues containing > 80% cancer cells were excised and used for ERα mRNA quantification. The ERα-status for the samples used in this study was determined either by IHC using monoclonal antibodies against NH_2_-terminal portion of the molecule at Oncotech Laboratories, Irwine, CA, or by ligand binding assay as described [10]. The tumor tissues were considered positive for ERα by IHC if > 5% of cancer cells showed positive nuclear staining. The tumor tissues that were diagnosed as ERα positive by estrogen binding assay had > 3 fmol of ER/mg of total tissue extract. A total of 70 samples positive by IHC, 33 positive by estrogen binding assay, 43 negative by IHC and 20 negative by estrogen binding assay were included in the current study (Tables 1, 2, 3 and 4 respectively). The tumor tissues were processed to isolate total RNA and cDNAs prepared as previously described [6-9]. Howard University Institutional Review Board granted the ethical approval of Tumor collection procedures for the study.

### Absolute quantification of ERα mRNA copy numbers by quantitative real-time PCR

Absolute quantification of ERα transcript copy numbers was achieved by quantitative real-time PCR in ABI Prism GeneAmp 7900 HT Sequence Detection System as described previously (9). Briefly, a typical real-time PCR reaction mixture contained cDNA prepared from reverse transcription of 0.5 – 5 nanograms of tumor tissue total RNA, 0.04 micromolar each of sense and anti-sense primers, 0.05 micromolar 5'FAM and 3'TAMARA labeled oligonucleotide probe and 1 × Taqman Universal PCR Mix in a total volume of 25 μl. PCR conditions were initial hold at 50°C for two minutes, followed by denaturation for ten minutes at 95°C, and denaturation for 15 seconds at 95°C in the subsequent cycles and annealing and extension for 1 min at 60°C for 40 cycles. The ERα mRNA copy numbers in tumor tissues were determined in comparison with a standard graph constructed simultaneously using 10^2^, 10^3^, 10^4^, 10^5^, 10^6^, 10^7^, 10^8^, and 10^9 ^copies of reverse transcribed cRNA of ERα. All the samples were amplified in triplicate and real-time PCRs were repeated four times. The ERα mRNA copy numbers in tumor tissues were normalized to mRNA copy numbers of the house keeping gene, glyceraldehyde 3- phosphate dehydrogenase (GAPDH). GAPDH copy numbers were determined as previously described [11,12]. The sense, and anti-sense primers and probe for quantifying the mRNA copy numbers of ERα were 5' caagcccgctcatgatcaa 3' (position, exon 4, bp 1110–1128), 5'ctgatcatggagggtcaaatccac3' (position, exon 5, bp 1358–1338) and FAM 5'agaacagcctggccttgtccctg3'TAMARA (position, exon 4, bp 1140–1162) respectively. The sense and anti-sense primers and probe for quantifying GAPDH mRNA copy numbers were 5'ttccagg agcgag atccct3' (position, bp 304–322), 5'ggctgttgtcatacttctcatgg3' (position, bp 483–505) and FAM 5' tgctggcgctgagtacgtcgtg3' TAMARA (position, bp 342–363) respectively. Primer positions of ERα and GAPDH nucleotide sequences were as described [13,14].

### Statistical analysis

Wilcoxon-rank-sum test and standard two-sample t-test were used to determine whether the mRNA copy numbers were significantly different in breast cancer tissues that were 1) ERα-positive and ERα-negative by IHC, 2) ERα-positive and ERα-negative by estrogen binding assays, 3) ERα positive by IHC and estrogen binding assays, and 4) ERα-negative by IHC and estrogen binding assays. Test results were considered significant if P ≤ 0. 05. To determine the cut-off value/maximum level of mRNA copy numbers in the samples which were negative by IHC and estrogen binding assays, we used CART (Classification Regression Tree) [15,16] program. The data consisting of 103 ERα positive and 63 ERα negative samples which had a predictive variable, mRNA copy numbers, were partitioned into two groups using CART program. This program determines the best cut-off value copy numbers in the sense that the OR (Odds Ratio, ERα positive to ERα negative in our case) of the two groups (with copy number greater than the cut-off value in one group and less or equal to the cut-off value in the other) is maximized. Receiver Operating Characteristic (ROC) analysis was performed to determine the sensitivity and specificity of the RNA based molecular assay. The ROC curves were generated by connecting all the points determined by the copy numbers in all the samples in an increasing order. Since data on IHC grading as percent positive cells were available on some of the samples, the correlation between the IHC grading and the mRNA copy number was determined both in the original scale and in logarithmic transformations scale using S-PLUS software.

## Results

We have undertaken the current study to determine ERα mRNA copy numbers in breast cancer tissues that were positive and negative by two conventional methods of assaying ERα protein, IHC and estrogen binding. The rational for undertaking this study is that once we establish a threshold value in IHC negative tissues, then the molecular assay could be applied in clinical laboratories in the place of currently used IHC assay for determining the status of ERα for prognostic and therapeutic purposes. Our rational for establishing a cut off value is that any patient who expresses above the cut off level could be selected as a candidate for anti-estrogen therapies and could be considered to have good prognosis.

**Figure 1:**
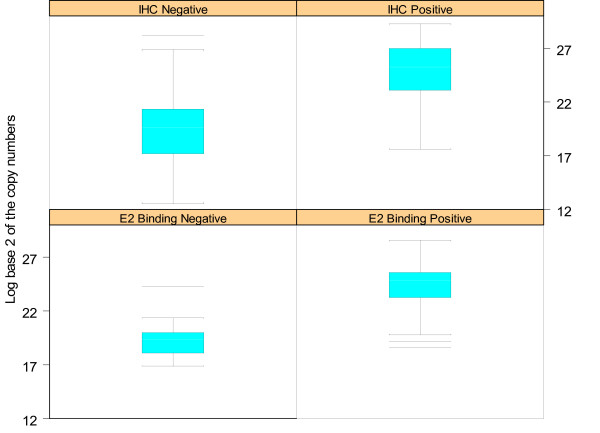
A Box plot drawn for the ERα mRNA copy numbers (logarithm base 2 scale) in the four groups (positive and negative by IHC and by estrogen binding assay) using S-PLUS software is shown.

We first profiled ERα mRNA copy numbers in 70 samples positive by IHC, 43 negative by IHC, 33 positive by estrogen binding assay and 20 negative by estrogen binding assay. The data are presented in Tables 1, 3, 2 and 4 respectively. A box plot drawn for the copy numbers (logarithm base 2 scale) in the four groups (positive and negative by IHC and by estrogen binding assay) using S-PLUS software is shown in Figure 1.

We next compared the quantitative data on mRNA copy numbers among samples as described below. 1) We tested whether the two conventional assays, the IHC and estrogen binding assays, correlate in terms of mRNA copy numbers in 70 and 33 positive tissues (Tables 1 and 2 respectively) using Wilcoxon-rank-sum test and standard two-sample t-tests. By these two tests, we did not find significant differences in the ERα mRNA copy numbers in samples that were ERα positive by IHC and estrogen binding assays (p > 0.28 by both tests). 2) We also compared the mRNA copy numbers in 43 samples negative by IHC with 20 samples negative by estrogen binding assay and did not find significant differences (Tables 3 and 4 respectively) (p > 0.25 by the above two tests). However, 3) we found significant differences in mRNA copy numbers in the breast tumors that were IHC positive from those which were IHC negative (Tables 1 and 3 respectively) (p = 1.3e-6 by standard two-sample t-test and p = 2.7e-18 by Wilcoxon-rank-sum test). And 4) we also found significant differences in the samples that were positive and negative by estrogen binding assay (Tables 2 and 4 respectively) (p = 7.6e-3 by standard two-sample t-test and p = 3.6e-7 by Wilcoxon-rank-sum test).

After establishing that ERα-positive tissues express significantly higher levels of mRNA copy numbers compared to negative tumor samples, we next determined the maximum level of expression in ERα-negative samples using CART program. By using this program, we found the maximum level of expression of mRNA copy numbers/cut-off value to be 5 × 10^6 ^per 10^10^copies of GAPDH mRNA. Of the total 106 positive (70 by IHC and 33 by estrogen binding assay) samples in our study, 83 samples showed higher level of expression than 5 × 10^6 ^copies per 10^10 ^copies of GAPDH. It is possible that the samples that showed less than the above cut off value copy numbers could be due to false positivity by the above methods. In a total of 63 negative samples (43 by IHC and 20 by estrogen binding assay) only 6 samples showed higher expression than 5 × 10^6 ^copies per 10^10^GAPDH copies. It is also possible that the samples that showed higher than the cut off copy numbers could be false negative. The OR (Odds Ratio) in the two groups is about 39.4, an extremely high OR value. The counts of the ERα-positive (80, 23) and ERα-negative (6, 66) in the two groups produce a chi-square value of 88.2544 with 1 degree of freedom, which is consistent with our T-test and Wilcoxon-rank-sum test results.

**Figure 2:**
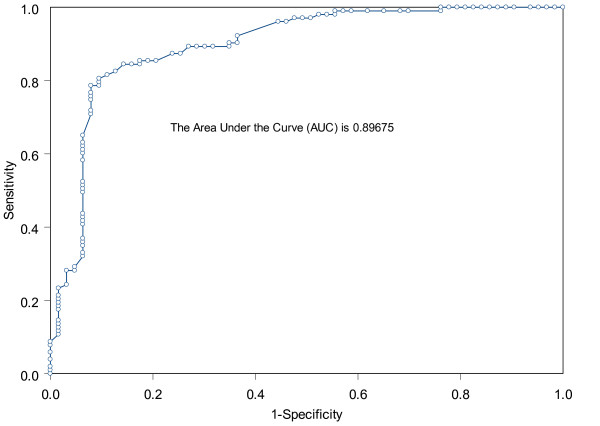
Receiver Operating Characteristic curve drawn using the data on ERα mRNA copy numbers is shown.

We applied the above cut off value and determined the Sensitivity (percentage of samples that showed higher copy numbers than the cut off value of 5 × 10^6 ^copies per 10^10 ^GAPDH copies in IHC positive tissues) and Specificity (percentage of samples that showed less than 5 × 10^6 ^copies per 10^10 ^GAPDH copies in IHC negative tissues) and the values obtained were 0.81 and 0.90 respectively. We also determined Receiver Operating Characteristics using S-PLUS software and the ROC curve generated is shown in Figure 2. The area under the ROC curve, 0.89675, shows that the molecular assay clearly distinguishes the positives by IHC or estrogen binding from the negative tissues.

Since we have the grading score as percent positive cells for about 50 samples (Table 1), we tested if a correlation exits between the percent positive cells by IHC and the mRNA copy numbers using S-PLUS software. We obtained a correlation coefficient of 0.02. When we used the logarithmic transformations scale, we obtained a correlation coefficient of 0.037. These results indicated that there is no correlation between the percent positive cells and the level of ERα mRNA copy numbers. These observations could be due to qualitative nature of IHC assay. The IHC data only show the number of positive cells but not quantitative to determine the level of ERα expression. The molecular assay based on RNA is quantitative to determine the level of ERα expression.

## Discussion and conclusion

Previously ERα mRNA levels in immunohistochemically positive and negative tissues were evaluated in breast cancer tissues by several groups using conventional RT PCR. Cullen et al [17] determined mRNA levels by conventional PCR in 107 breast cancer tissues. They reported that ERα mRNA was more frequently detected in ERα protein positive tissues than ERα protein negative tissues. Jarzabek et al [18] studied ERα mRNA levels and protein levels in 41 primary breast cancer tissues. They reported the presence of ERα mRNA in all the tissues, where as the protein was present only in 70% of tumor tissues by Western blotting and 67% showed positive by immunohistochemistry. They concluded that lack of ERα protein is not due to lack of ERα gene expression or methylation of its promoter, but may be due to post-transcriptional or post-translational mechanisms. Alkarain et al [19] reported the presence of ERα mRNA in immunohistochemically ERα-negative tissues. However, none of the above studies has evaluated the threshold levels of ERα mRNA levels in immunohistochemically negative tissues or those negative by ligand binding assays. Our quantitative analysis of ERα mRNA copy numbers demonstrate that breast cancer tissues that are negative by both IHC and ligand binding express significant levels of ERα mRNA. The reasons why the mRNA is not translated to detectable protein are not clear. Our previous studies on ERβ mRNA copy numbers [9] in breast cancer tissues have shown that at the 5 × 10^6 ^copies per 10^10 ^mRNA copies of GAPDH levels ERβ protein is translated. It is possible that either ERα mRNA is not translated or the translated protein is degraded to undetectable levels in these tissues.

The results and the analysis presented above clearly demonstrate that the ERα positive tissues by IHC or estrogen binding assay express significantly higher mRNA copy numbers than 5 × 10^6 ^copies per 10^10 ^GAPDH copies. An extremely high Odds Ratio, high sensitivity and specificity demonstrate that the molecular assay could be used in the place of currently used IHC in the clinical laboratories. Based on our data above any patient who has more than 5 × 10^6 ^copies per 10^10 ^GAPDH copies in her tumor tissue could be considered positive for ERα, could be selected for anti-estrogen therapies and considered to have good prognosis. However, the above described approach has some limitations in that it needs to be verified on a defined set of biopsy samples and with reference to another house keeping gene. Therefore, the results should be interpreted with caution and undoubtedly will require confirmation by a larger prospective multi-centered clinical study with a more accurate design to bring the technology to the clinic. The current study is a first step in that direction. We expect that the cost effective, extremely sensitive, high though-put molecular assay which requires only a few cancer cells could be an assay of choice to replace IHC in clinical labs for determining ERα status in breast cancer tissues once established in a multi-centered prospective clinical study.

## Abbreviations

FAM, carboxy-fluorescein; TAMARA, 6-carboxy tetraethyl-rhodamine; GAPDH, Glyceraldehyde-3 phosphate dehydrogenase; ERα, estrogen receptor alpha; IHC, Immunohistochemistry; CART, Classification And Regression Tree; and OR, Odds Ratio and ROC, Receiver Operating Characteristics.

## Competing interests

The author(s) declare that they have no competing interests.

## Authors' contributions

I. P conceived the study, participated in the design of the study, performed the real-time PCR quantifications of ERalph mRNA copy numbers and drafted the manuscript. Q.Y participated in performing the statistical analysis of the data. Both authors read and approved the final manuscript.

## Pre-publication history

The pre-publication history for this paper can be accessed here:



## Figures and Tables

**Table 1:** ERα mRNA copy numbers in breast cancer tissues that were positive by IHC assay

**#**	**Grading by IHC (Percent of positive cells)**	**ERα mRNA copy numbers per 10^10 ^mRNA copies of GAPDH**
1	100	1.50e^8^
2	100	6.50e^6^
3	100	1.33e^8^
4	100	1.00e^8^
5	100	3.00e^8^
6	100	2.20e^7^
7	100	2.50e^7^
8	100	6.50e^8^
9	100	2.00e^5^
10	99	2.25e^8^
11	98	1.33e^8^
12	98	7.14e^7^
13	98	4.50e^8^
14	97	4.50e^8^
15	95	8.00e^7^
16	91	2.06e^8^
17	90	5.71e^7^
18	90	2.50e^8^
19	90	3.80e^7^
20	90	2.08e^8^
21	90	4.50e^7^
22	90	8.50e^7^
23	90	2.50e^6^
24	90	1.88e^8^
25	90	1.80e^7^
26	90	1.25e^8^
27	90	2.86 e^7^
28	90	1.00 e^8^
29	90	3.00e^7^
30	90	4.17e^6^
31	90	3.75e^8^
32	90	8.50 e^6^
33	90	1.70e^8^
34	90	1.13e^6^
35	90	2.00e^7^
36	90	3.00e^7^
37	90	5.00e^7^
38	90	4.25e^7^
39	80	2.86e^7^
40	80	2.50e^6^
41	80	2.83e^7^
42	80	4.88e^7^
43	60	3.25e^7^
44	60	2.00e^6^
45	40	1.00e^6^
46	40	1.35e^8^
47	30	5.00e^8^
48	20	8.00e^7^
49	*+	2.06e^7^
50	*+	6.06e^7^
51	*+	4.0e^7^
52	*+	8.0e^7^
53	*+	5.0e^6^
54	*+	2.06e^7^
55	*+	3.06e^7^
56	*+	4.06e^8^
57	*+	6.06e^7^
58	*+	1.06e^6^
59	*+	4.06e^8^
60	*+	1.06e^6^
61	*+	8.0e^6^
62	*+	2.0e^6^
63	*+	3.0e^7^
64	*+	2.0e^8^
65	*+	1.0e^6^
66	*+	7.0e^6^
67	*+	2.0e^7^
68	*+	4.0e^7^
69	*+	8.0e^6^
70	*+	9.0e^6^

* These samples were collected at the time when the practice was to grade the samples as just Positive or Negative but no numerical scorings were given.

**Table 2:** ERα mRNA copy numbers in breast cancer tissues that were graded as positive by estrogen binding assay

**#**	**ERα by ligand binding**	**ERα mRNA copy numbers per 10^10 ^mRNA copies of GAPDH**
1	+	9e^5^
2	+	5e^7^
3	+	3e^7^
4	+	4e^7^
5	+	2e^7^
6	+	6e^5^
7	+	4e^6^
8	+	4e^8^
9	+	2e^7^
10	+	2e^7^
11	+	1e^7^
12	+	1e^7^
13	+	1e^8^
14	+	1e^7^
15	+	4e^5^
16	+	4e^6^
17	+	4e^7^
18	+	1e^6^
19	+	4e^7^
20	+	4e^8^
21	+	6e^7^
22	+	2e^8^
23	+	5e^7^
24	+	4e^7^
25	+	1e^6^
26	+	3e^7^
27	+	3e^7^
28	+	3e^6^
29	+	2e^8^
30	+	2e^7^
31	+	1e^8^
32	+	3e^8^
33	+	5e^7^

**Table 3:** ERα mRNA copy numbers in breast cancer tissues that were negative by IHC

**#**	**Grading by IHC**	**ERα mRNA copy numbers per 10^10 ^mRNA copies of GAPDH**
1	-	1.25e^6^
2	-	1.25e^6^
3	-	1.00e^5^
4	-	3.75e^6^
5	-	8.00e^5^
6	-	2.00e^6^
7	-	3.00e^5^
8	-	2.50e^6^
9	-	2.50e^5^
10	-	6.25e^4^
11	-	1.67 e^6^
12	-	5.00e^4^
13	-	5.00e^5^
14	-	1.75e^5^
15	-	4.17e^6^
16	-	2.50e^5^
17	-	2.65e^6^
18	-	2.50e^6^
19	-	1.25e^5^
20	-	1.17e^4^
21	-	9.00e^5^
22	-	3.75e^5^
23	-	6.00e^3^
24	-	1.50e^5^
25	-	3.75e^4^
26	-	2.67e^5^
27	-	3.20e^5^
28	-	1.67e^5^
29	-	1.40e^6^
30	-	4.00e^6^
31	-	3.00e^4^
32	-	3.00e^5^
33	-	1.00e^6^
34	-	1.25e^8^
35	-	3.33e^6^
36	-	8.00e^7^
37	-	7.50e^6^
38	-	9.00e^7^
39	-	5.00e^6^
40	-	3.00e^8^
41	-	1.17e^5^
42	-	5.00e^4^
43	-	1.00e^6^

**Table 4:** ERα mRNA copy numbers in breast cancer tissues that were negative by estrogen binding assay

**#**	**ERα by ligand binding**	**ERα mRNA copy numbers per 10^10 ^mRNA copies of GAPDH**
1	-	1.6e^5^
2	-	2.8e^5^
3	-	5.6e^5^
4	-	2.5e^5^
5	-	3.2e^5^
6	-	2.8e^5^
7	-	1.2e^5^
8	-	3.3e^5^
9	-	2.5e^5^
10	-	1.5e^6^
11	-	2.7e^6^
12	-	1.0e^6^
13	-	2.0e^7^
14	-	1.0e^6^
15	-	9.5e^5^
16	-	2.1e^6^
17	-	6.0e^5^
18	-	1.0e^6^
19	-	1.1e^6^
20	-	7.7e^5^
